# Follow up colonoscopy may be omissible in uncomplicated left-sided acute diverticulitis diagnosed with CT- a retrospective cohort study

**DOI:** 10.1038/s41598-019-56641-2

**Published:** 2019-12-27

**Authors:** Nollaig O’Donohoe, Pankaj Chandak, Marina Likos-Corbett, Janelle Yee, Katherine Hurndall, Christopher Rao, Alec Harry Engledow

**Affiliations:** Department of Surgery, Queen Elizabeth Hospital, Woolwich, London, UK

**Keywords:** Health care economics, Colonoscopy

## Abstract

International guidelines recommend colonoscopy following hospitalisation for acute diverticulitis. There is a paucity of evidence supporting the efficacy of colonoscopy in this context, particularly for patients with CT-diagnosed uncomplicated left-sided diverticulitis. This study aims to investigate the frequency that colorectal cancer (CRC) and advanced adenomas (AA) are identified during follow-up colonoscopy after hospitalisation with CT-proven left-sided diverticulitis for the first time in a UK population. In this single-centre retrospective-cohort study all patients presenting with CT-diagnosed uncomplicated left-sided diverticulitis between 2014 and 2017 were identified. The incidence of histologically confirmed CRC and AA identified at follow-up colonoscopy 4–6 weeks following discharge was assessed. 204 patients with CT proven uncomplicated left-sided diverticulitis underwent follow-up colonoscopy. 72% were female and the median age was 63 years. There were no major complications. 22% of patients were found to have incidental hyperplastic polyps or adenomas with low-grade dysplasia. No CRC or AA were found. Routine colonoscopy following acute diverticulitis in this cohort did not identify a single CRC or AA and could arguably have been omitted. This would significantly reduce cost and pressure on endoscopy departments, in addition to the pain and discomfort that is commonly associated with colonoscopy.

## Introduction

In UK, as with the rest of the developed world, diverticulitis is an increasingly frequent presentation to the emergency department^1,2^. Up to one quarter of patients with diverticulosis will require hospital management of their acute diverticulitis at one point in their lives^3^. The average age at presentation is falling, compounding the clinical burden of this condition and raising more controversy around the optimal management of acute diverticulitis^4–7^. The management of uncomplicated diverticulitis is undoubtedly moving away from surgery^6,8,9^. The value of an invasive, uncomfortable, risk associated procedure such as follow up colonoscopy also needs to be justified^10^.

Long established international guidelines recommend follow up colonoscopy to exam for cancer in all patients post an episode of acute diverticulitis^11–14^. This is the position taken by both the Royal College of Surgeons of England and the Association of Coloproctology of Great Britain and Ireland^15,16^. The guidelines of the American Society of Colon and Rectal Surgeons recommend similar^17^. Emerging evidence, however, appears to refute the expert opinions on which these guidelines are based^6,18–23^. As present day CT scanners have a sensitivity of 94% and a specificity of 99%, the gain from a follow up colonoscopy after a CT diagnosis of left-sided uncomplicated diverticulitis is questionable^10,24^.

There is a paucity of UK based studies challenging the diagnostic value of colonoscopy following uncomplicated left-sided acute diverticulitis. With increasing pressure on NHS resources it is important to examine the economic value as well as the clinical benefit of follow up colonoscopy. In this study we aim to evaluate the frequency at which colorectal cancer (CRC) and advanced adenomas (AA) are detected during routine colonoscopy following an acute episode of acute left sided diverticulitis in a UK district general hospital^25^.

## Method

All patients over the age of 18 with CT-diagnosed uncomplicated left-sided diverticulitis, admitted 2014–2017, with a follow-up colonoscopy 4–6 weeks after admission were eligible for this study. All hospital admissions with suspected acute diverticulitis were identified by retrospective review of the surgical department’s computer based emergency admission record. Only the cohort of diverticulitis patients with a CT-scan reporting left sided diverticulitis with a modified Hinchey classification of 0 or 1a were included^25,26^. All CT scans were reported by a consultant radiologist. Demographic, endoscopic and histological data on this cohort was then collected from the hospital’s main computer database. All colonoscopies were carried out or supervised by Joint Advisory Group for Endoscopy approved endoscopists. The hospital endoscopy database (ADAM, Japan) was interrogated to ensure all colonoscopies met JAG quality indicators. All patients with endoscopic findings requiring biopsy or polypectomy were identified and the histology from these patients reviewed. As with a well-executed Dutch study published in Surgical Endoscopy in 2015, the primary outcome was the incidence of histologically confirmed colorectal carcinoma diagnosed on follow-up colonoscopy and the secondary outcome was the incidence of advanced adenoma^25^.

The study intentionally excluded all patients with complicated diverticulitis, as multiple studies have shown the value of follow-up colonoscopy in these patients^27^. As the incidence of left sided diverticulitis is far higher than right sided, we excluded any patients with right sided diverticulitis^1^. Patients who had follow-up CT rather than colonoscopy and those with a colonoscopy within a year before presentation were excluded^27^.

This retrospective cohort study was registered and approved by the research and development department in Lewisham and Greenwich NHS Trust. All local ethical and information governance standards were fully met. Informed consent was given by all patients for the procedures undertaken. The datasets generated during and/or analysed during the current study are available from the corresponding author on reasonable request.

## Results

**Figure 1:**
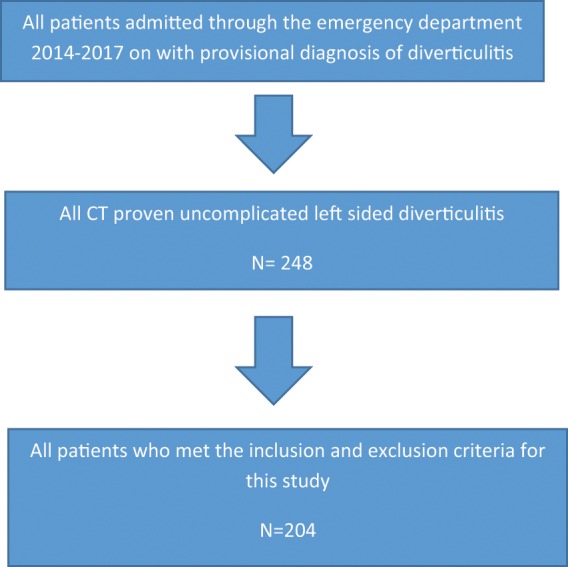
Flow diagram summarising identification and exclusion of trial participant.

248 patients were diagnosed with acute uncomplicated left-sided diverticulitis on CT scan. After discharge from hospital 204 patients of these patients underwent follow up evaluation by colonoscopy and were included in this study (Fig. 1). Of the patients who underwent follow-up colonoscopy 146 (72%) were female and the median age was 63 years (Range 29–90 years). The median length of stay was 3 days (Range 3–5 days). The median number of days to follow up colonoscopy was 37 (Range 27–68 days). There were no major complications following colonoscopy. 45 (22%) of patients were found to have incidental polyps and all of these were histologically benign. 14 (31%) of the polyps found were hyperplastic and 31 (68%) were adenomas with low grade dysplasia. No CRC were found (Tables 1 and 2).

**Table 1:** Endoscopic findings.

Endoscopy findings	Number of Patients
Normal Examination	0
Diverticulosis	204
Stricture	0
Polyps	45
Colorectal Cancer	0

**Table 2:** Histological findings of excised polyps.

Polyp Histology	Number of Patients
Hyperplastic	14
Inflammatory	0
Low grade dysplasia	31
High grade dysplasia	0

## Discussion

This study demonstrated no increased diagnostic gain in performing a follow up colonoscopy on uncomplicated left-sided acute diverticulitis patients confirmed with CT. There was no CRC nor was there any histologically confirmed AA found in the cohort of 204 patients.

Colonoscopy is labour intense, costly and not without risk^28^. 1 in 2000 patients have bleeding requiring intervention and 1 in 2500 colonoscopies result in perforation^29^. At approximately £450 per colonoscopy, omission of endoscopic follow up would be a significant cost saving measure for our hospital^30^. Patients can still avail of the national bowel screening programme when they reach 50 years of age^25^.

Multiple studies have questioned the continued need for follow up colonoscopy when acute uncomplicated left sided diverticulitis has been diagnosed with multi-slice CT imaging. In 2011 Westwood *et al*. published a retrospective longitudinal study of patients with acute uncomplicated diverticulitis diagnosed with CT in the British Journal of Surgery^19^. There were 292 patients diagnosed with uncomplicated left-sided acute diverticulitis on CT, of which 205 went on to have colonoscopy. Colorectal cancer was picked up at a similar rate to the average population undergoing screening. Based on these findings the authors concluded that in uncomplicated left-sided acute diverticulitis follow up colonoscopy may be unnecessary. A 2014 systematic review and meta-analysis of 1497 patients analysed the results of follow up colonoscopy after a CT diagnosis of acute diverticulitis^31^. Colorectal cancer was found in 5 of the 1497 patients and the authors again concluded follow up colonoscopy may not be necessary. These findings were corroborated by a 2015 Dutch paper^25^. This study compared the colonoscopic detection rate of AA and CRC in 401 patients diagnosed with left sided uncomplicated acute diverticulitis on CT, with 1426 patients in a colorectal screening programme. The AA and CRC detection rates were alike between the two groups and the paper recommended the omission of follow-up colonoscopy after CT diagnosed uncomplicated left-sided acute diverticulitis. The EAES and SAGES 2018 consensus conference on acute diverticulitis management recommended against follow up colonoscopy in uncomplicated left-sided acute diverticulitis^6^.

In our retrospective cohort study, colonoscopic detection rates of CRC and AA after uncomplicated acute left-sided diverticulitis are comparable to published research. This challenges current UK guidelines and suggests that it is reasonable to omit follow-up colonoscopy after an episode of CT-diagnosed uncomplicated left-sided acute diverticulitis. The implications in our hospital and more broadly in the NHS would be a reduction in costs and burden on endoscopy departments. It would also avoid the pain and discomfort incurred by patients undergoing colonoscopy. Arguably it could also reduce the small but important risk of endoscopic complications.

A number of limitations are acknowledged in our study methodology. This was a retrospective, single-centred study. Advancing age is a recognised risk factor for colorectal cancer and our median age was only 63 years^32^. The study, however, included a well-defined previously unstudied cohort of patients. All radiological reporting was at consultant level and only patients whose colonoscopies met JAG quality indicators were included.

In conclusion, this retrospective cohort study suggests that follow up colonoscopy may be omissible in CT-diagnosed uncomplicated left sided diverticulitis.
